# Multiple Sclerosis Impact on Pregnancy: An Update on Management Issues: Management of MS During Pregnancy

**DOI:** 10.31661/gmj.v11i.2703

**Published:** 2022-12-31

**Authors:** Ahmadreza Badali, Rahil GhorbaniNia, Shima Mohammadian, Sahar Poudineh, Alireza Sarlak, Mohammadreza Eghbali, Esmaeil Behzadi, Morteza Jafarinia

**Affiliations:** ^1^ Medical School, Tabriz University of Medical Sciences, Tabriz, Iran; ^2^ Faculty of Management and Medical Information, Kerman University of Medical Sciences, Kerman, Iran; ^3^ Department of Gynecology Oncology, Kamali Teaching Hospital, Alborz University of Medical Sciences, Karaj, Iran; ^4^ School of Medicine, Mashhad Azad University, Mashhad, Iran; ^5^ School of Medicine, Hamedan University, Hamedan, Iran; ^6^ School of Medicine, Shiraz University of Medical Sciences, Shiraz, Iran; ^7^ Shiraz Neuroscience Research Center, Shiraz University of Medical Sciences, Shiraz, Iran

**Keywords:** Multiple Sclerosis, Pregnancy, Disease-Modifying Treatments, Relapsing-remitting, Primary Progressive

## Abstract

Women with multiple sclerosis (MS) of reproductive age are becoming pregnant at an alarmingly high rate. Disease control is required during the preconception, prenatal, and postpartum periods to reduce the likelihood of relapses of MS while minimizing hazards to the mother and fetus. It has long been understood that the disease activity of MS noticeably decreases in the third trimester of pregnancy, then noticeably increases in the first three months after delivery before returning to its pre-pregnancy baseline. Relapse during pregnancy and high rates of relapse before becoming pregnant have both been linked to an increased risk of postpartum attacks. In patients with relapse MS, recent results continue to support the notion that pregnancy does not affect long-term disease progression (and may even have the opposite effect); the situation is less clear for patients with progressive MS. It is comforting to know that none of the MS disease-modifying medications have been shown to cause teratogenic consequences. This review discusses the effects of pregnancy on disease activity and how to handle relapses when pregnant and breastfeeding.

## Introduction

Multiple sclerosis (MS) is a chronic autoimmune and degenerative disease affecting the central nervous system (CNS), which first manifests as demyelination, inflammation, and axonal destruction [1]. In a healthy population, the prevalence of MS is 1:1,000; however, when one twin has MS, the risk is one in four for identical twins [2][3]. For those with a genetic predisposition to MS, physical and mental stress, recurrent exposure to chemical solvents, ultraviolet radiation, and Epstein-Barr virus infection are thought to be the main risk factors [4][5]. Relapsing-remitting MS (RRMS) is the most common form of MS, but primary progressive MS (PPMS), which occurs in about 15% of individuals, is also a common presentation [6]. MS mainly affects women who are ready to have children (aged 20-40 years). Decades ago, records from the seminal pregnancy in MS (PRIMS) study disproved theories that pregnancy might be hazardous for MS women [7]. The PRIMS trial also showed a higher chance of relapses in the first three to four months following birth, particularly in patients whose MS was very active before becoming pregnant [7]. Before 50 years, 90% of individuals experience symptoms, and one in three female patients diagnosed with MS become pregnant [8]. Due to the potential negative effects of the various disease-modifying treatments (DMTs) for the fetus and the woman before or after conception, the rising of MS incidence among women in their reproductive age continues to be a clinical concern [9]. Disease control care is required during the preconception, prenatal, and postpartum periods to reduce the likelihood of relapses of MS while minimizing hazards to the mother and fetus [10].

The current study aimed to review the literature on the data available regarding the role of pregnancy on disease activity, concerns before becoming pregnant, accessible DMTs, and management relapses throughout pregnancy.

## The Immune System During the Pregnancy

In contrast to popular belief, pregnancy is thought to be an immunotolerant state in which the maternal immune system adjusts to an allogeneic pregnancy [11]. To create fetomaternal microchimerism, the mother and fetus actively communicate while exchanging cells in both directions [12]. Pregnancy brings about four significant biological changes that are likely relevant for MS. First, levels of a variety of hormones, including estrogen (particularly estriol), progesterone, prolactin, and glucocorticoids rise noticeably during pregnancy and then sharply decrease after delivery. These hormones greatly impact the immune system. They alter cytokines, lessen matrix metalloproteinases and adhesion molecules, reduce antigen presentation, and increase regulatory T-cell counts (T-regs) [11]. In the end, inflammatory processes are reduced. Second, natural killer cells, T-helper 17 cells, and T-regs undergo substantial changes during pregnancy. Third, the maternal immune system is affected by and interacts with fetal antigens. There is fetal antigen-specific peripheral T-regs present [13]. Maternal dendritic cells (highly effective antigen-presenting cells) pick up fetal antigens secreted into tiny micro vesicles. Finally, new studies indicate that pregnancy may help the maternal CNS by encouraging endogenous recovery mechanisms and improving the capacity to react to immune-mediated harm [14]. It is hoped that understanding how the immune system functions during pregnancy could inspire new MS treatment approaches.

## Pre-pregnancy Considerations

Pregnant patients worry about a variety of things, including the mother's capacity to breastfeed and raise the kid, fertility, the influence of pregnancy on MS activity, the likelihood of MS transmission to the unborn child, and the effects of medications on pregnancy [8]. The fact that MS is thought not to affect a woman's capacity to engender and bear a child to term, as well as the fact that the diagnosis of MS does not raise the likelihood of preterm or stillbirth, birth abnormalities, spontaneous abortions, and cesarean delivery should reassure women [15]. Patients with MS are assumed to have the same potential to conceive as healthy persons, despite women with MS having fewer pregnancies before the first MS attacks [16]. Case-control research was conducted to examine the anti-Müllerian hormone (AMH) levels in patients with MS and healthy people [17]. AMH levels, a measure of ovarian reserve, were considerably lower in MS patients than in the control group [17]. Sexual dysfunction is rather prevalent among MS sufferers. In a study, 61% of men and 63% of women reported that their MS made it difficult to engage in sexual activity [18]. Patients with infertility rates of around 10% may use assisted reproductive procedures, such as in vitro fertilization (IVF), with success rates of up to 39% in the group of women under 35 years [19][20]. The probability of clinical and/or magnetic resonance imaging (MRI) disease activity for three months may be greater in women who underwent IVF but could not conceive [15]. Gonadotropin-releasing hormone (GnRH) agonist use is associated with a higher incidence of relapses. Pro-inflammatory cytokines such as interferon-γ, interleukin (IL)-12, IL-8, and transforming growth factor-β are produced more often by GnRH [20]. Chemokine ligand12 and vascular endothelial growth factor (VEGF) are also present in higher concentrations, which facilitates the passage of mononuclear cells across the blood-brain barrier (BBB) [21]. Due to the importance of VEGF and VEGF-enhancing factor production in CNS angiogenesis during MS development, this pathway might lead to higher risks of MS relapses following fertility therapy. Endothelin-1 and angiopoietin-2 concentrations in the blood serum of MS patients are significantly raised and are thought to boost VEGF's angiogenic response [21]. Vitamin D supplements should be recommended to all women trying to get pregnant. In addition, they also advise eliminating smoking, quitting alcohol, and taking folic acid and prenatal vitamins [22]. There is evidence from numerous research that there is a correlation between lower serum vitamin D concentrations and MS activity, pointing to vitamin D insufficiency as a risk factor for MS development and increased disease activity [23]. Because of this, pregnant women with vitamin D deficiency had a 2-fold higher risk of MS than the control group [23]. According to research, having enough vitamin D during pregnancy may reduce the risk of MS developing in the offspring [24]. MS patients worry differently about the possibility of passing the illness on to children. A decision not to have children was made by 34.5% of MS patients, according to the MS database maintained by the North American Research Committee [25].

## Impacts of MS on Pregnancy

Regarding the normal course of MS during pregnancy, the PRIMS research, which was published in 1998, continues to be the standard [26]. The major findings were supported by related investigations conducted later [27]. The annualized recurrence rate (ARR) considerably decreased during pregnancy, notably in the third trimester, compared to the pre-pregnancy year in a cohort study of 254 MS patients who were prospectively monitored during 269 pregnancies between 1992 and 1995 [26]. For 28% of the women, the first three months following delivery was a resurgence of disease activity. Notably, the pregnancy-overall year's ARR (nine months of pregnancy plus three months after delivery) was similar to the previous year [26]. The postpartum rebound appeared to be less noticeable in more recent investigations, and this finding may be related to DMT exposure before conception. According to studies, DMT use before becoming pregnant may lessen the likelihood of postpartum relapse, contradicting an Italian study in which no connection was detected; however, 46.4% of the women had used a DMT before becoming pregnant [27][28]. Uncertainty over how exposure months before birth may affect postpartum relapse may justify continuing DMT use as long as possible, if not until conception when there is no chance of fetal harm. Since twenty years ago, a vast majority of women with RRMS have been treated, and the arsenal of medications available to prevent relapses has significantly expanded [27]. As a result, it impacts the clinical course both throughout pregnancy and after delivery. Hughes *et al*. monitored nearly 900 pregnancies, and their findings indicate that using DMTs before conception was related to a 45% lower risk of postpartum relapse in the first three months following birth [27]. These findings were corroborated in two further retrospective cohorts with 152 and 99 pregnancies [28]. Even in the most recent period, secondary progressive MS and PPMS are extremely uncommon phenotypes during pregnancy due to the typical age at which conception occurs [28]. A recent MS Base survey conducted in 33 countries revealed 1178 pregnant women and 1521 pregnancies in total [29]. Except for eight patients who had PPMS, every patient had RRMS. When disability and geographic location were taken into account, adjusted analyses revealed that age and the likelihood of pregnancy were not significantly correlated [29]. A lower pregnancy incidence was linked to greater Expanded Disability Status Scale (EDSS) scores, with a relative incidence risk ratio of 0.96 per EDSS point. Clinical experience makes it clear that receiving an MS diagnosis affects the decision to have children. However, there is not much research on how often MS-affected women become pregnant. A recent retrospective study compared 58 million women without MS to a 5% random sample to examine pregnancy and its outcome between 2006 to 2015 [30]. The adjusted proportion of pregnant women with MS grew from 7.9 to 9.5% between 2006 and 2014, while the proportion of pregnant women without MS concurrently fell from 8.8 to 7.7%, both trends being statistically significant [30]. Women in the Danish MS Registry who were diagnosed with MS between 1960 and 1996 were matched to four reference individuals [31]. Women with MS generally had fewer children from 1960 to 2016, with an incidence rate ratio of 0.63; however, this was equally true for men [31]. It is intriguing to note that since the 1990s, the proportion of live births following diagnosis has risen for both women and men. Benoit *et al*. examined the risk of recurrence during pregnancy and after birth in 93 French and Italian women who had two subsequent pregnancies following the beginning of MS [32]. Only 7.6% of women relapsed after both pregnancies, while most women didn't in the three months after giving birth. Although the relapse rates were slightly lower during the second pregnancy, the overall course of MS relapse was similar in both pregnancies [32]. In subsequent pregnancies, there was no association between exercise levels. Therefore, the counseling provided to women who intend to become pregnant again should be the same as it was for their first pregnancy [32]. The long-term impact of childbirth on the development of disabilities is another crucial aspect of prenatal counseling. In a study, 330 women with MS were divided into four groups; without children, with children both before and after MS beginning, and with children only before or after MS onset [33]. D'hooghe *et al*. compared the time from disease onset to reaching EDSS equal to six. They discovered that the progression of impairment was much slower in women who had children after the onset of MS [33]. This result was unquestionably complicated by the disease's initial severity, which forced women to decide whether or not to become pregnant and subjected analyses to an eternal time bias [33]. The Barcelona group recently attempted to resolve this problem using contemporary statistical techniques, including propensity matching. They demonstrated in 501 women that when pregnancy was taken into account as a baseline variable, it was related to a beneficial outcome, but this association vanished when pregnancy was taken into consideration as a time-dependent variable [34]. Pregnancy is therefore probably not harmful or at least does not affect how a disability develops [35].

## Risk for MS in Children

The risk of developing MS among children is roughly 2% in a situation where one or both parents have MS, and between 6% and 12% of children with both parents' MS have been reported (congenital MS) [36]. Counseling should emphasize that a child's chance of developing MS is likely years away, that it could have a benign course, and that ongoing advancements in MS research include creating more efficient treatments [36]. Although the year and place of birth may influence the results, minor changes in the risk of MS according to the month of birth (greater in spring, lower in autumn) were reported [37]. MS risk is lower among the offspring of mothers who consumed more vitamin D during pregnancy, which has been linked to lower vitamin D levels and a higher risk for the disease [38]. Supplementing mothers who lack vitamin D may seem sensible [38], but even the safety of vitamin D supplementation during pregnancy has not been proven. However, the dose should reach and not exceed a normal serum concentration [39].

## Pregnancy and MS Activity

The onset of MS during pregnancy is unusual. Relapse can happen less frequently than when the patient is not pregnant [26]. The last trimester of pregnancy significantly lowers MS disease activity. Based on the PRIMS research, 227 pregnancies, each of which resulted in a live delivery, were included for analysis, and patients were monitored for at least a year after delivery [26]. The annualized recurrence rate decreased by 70% throughout the third trimester compared to pre-pregnancy; it increased to 70% above the pre-pregnancy level during the first three months after delivery, then decreased and remained at the pre-pregnancy rate [26]. Multiple prospective clinical trials have validated this suppression of clinical episodes in later pregnancy, and much more sparse MRI data demonstrating a slower accumulation of quiet brain lesions supports this claim. In a study of 19 MS-afflicted women, postpartum activity was verified, with 11 (58%) showing an increase in postpartum MRI lesion activity compared to third-trimester activity [40]. The three-month postpartum period of increased risk has also been consistently noted. During this time, about 30% of MS patients experience relapses. There have been attempts to pinpoint the predisposing elements for postpartum attacks. High relapse rates in the year before conception, a higher level of disability before conception, and relapse during pregnancy have all been repeatedly found to be predictors of postpartum activity [41]. In Ponsonby *et al*. study, 282 women with the clinically isolated syndrome (CIS) were compared with 542 matched controls; more pregnancies and births were associated with a lower risk of having such a first incident, which is consistent with a cumulative protective impact of pregnancy [41]. Radiologically isolated syndrome (RIS) is the finding of a severely aberrant brain MRI that is suggestive of MS in otherwise clinically healthy people [42]. It can be a sign of asymptomatic or early-stage MS. Seven of the 60 women in the study who had RIS got pregnant [42]. Pregnancy was linked to increased clinical and MRI disease activity in this small RIS cohort, suggesting that a silent disease had become active [42]. More research on CIS and RIS is required to further explain these ideas.

## Treatments During Pregnancy

The use of medication by pregnant women is always a risk. The United States Food and Drug Administration (FDA) has only seldom given pregnant medications their highest rating, category A. Treatment issues for MS patients can be categorized into two categories; usage of DMTs and symptomatic therapy [43].

### 1. DMTs

#### 1.1. Interferons-β (IFN-β)

IFN-β is a polypeptide with a molecular weight that varies from 18.5 to 22.5 kDa; as a result, IFN-β cannot enter the placenta due to its high molecular weight [44]. The European Medicines Agency (EMA) published an update in September 2019 that allowed its users to be considered before conception, during pregnancy, and during nursing [45]. The updated advice was based on safety information from two cohort studies looking at pregnancies affected by IFN-β. The European IFN-β Pregnancy Registry examined 948 pregnant women in 26 countries and found no appreciable differences in spontaneous abortion or inborn abnormality rates between the general population and women exposed to IFN-β before and/or during pregnancy [45]. Another comparable Nordic cohort study detailing the outcomes of 411 Swedish and 232 Finnish pregnancies exposed to IFN-β showed that infants treated with IFN-β during pregnancy did not vary from the unexposed group in terms of birth weight, length, or head circumference [46]. IFN-β gets into breast milk in minimal amounts during breastfeeding (around 0.006% of the maternal dose) due to its large molecular weight [47]. Thirty-nine breastfeeding women were examined in a just-completed study by Ciplea *et al*. under the influence of IFN-β. In the first year of life, potential newborn exposure to IFN-β had no adverse effects [48].

### 1.2. Teriflunomide

In pregnant rats, teriflunomide significantly increases the fetal risk abnormalities and embryofetal mortality [49]. Teriflunomide is the only DMT that should be considered for males who are receiving treatment because it also enters semen. Leflunomide, an oral medication used to treat rheumatoid arthritis for many years, has an active metabolite called teriflunomide. Leflunomide exposure during pregnancy has not been linked to teratogenic effects in the limited experience [49]. The clinical development program for teriflunomide has also not found any evidence of teratogenicity in humans [50]. Before initiating treatment, pregnancy should be ruled out. Teriflunomide seems to stay in the body for 24 months, but a washout program can eliminate it [51]. Teriflunomide is produced in breast milk after crossing the placenta in the rat model [51].

### 1.3. Glatiramer Acetate (GA)

Similar to IFN-β, GA is a polypeptide with a high molecular weight, and it is unlikely that it could breach the placental barrier. Data from substantial research reviewing Teva's global pharmacovigilance database, which includes 7000 pregnancies gathered over 20 years, provided important information on the safety of GA in pregnancy. There were 5042 pregnancies with known outcomes in total, including 4034 live births (2366 healthy infants, 1557 newborns without inborn abnormalities, and 111 newborns with congenital and/or perinatal disruptions); 138 elective abortions; 53 extrauterine pregnancies; 49 stillbirths; 9 pregnancy terminations without a specific reason; and six newborns with hydatidiform moles [52]. Trisomy 21, cardiac abnormalities, talipes equinovarus, and hip dysplasia were the most common congenital malformations [52]. The study concluded that there was no higher incidence of inborn defects in fetuses exposed to GA when these results were contrasted to the general population [52]. This information proved that GA exposure during pregnancy seems safe and does not cause teratogenic effects [52]. Most experts believe GA is safe to use during breastfeeding; however, little research supports this. Thirty-four breastfeeding mothers who received GA daily were followed for one year; however, no symptoms linked to possible GA exposure in infants were observed [48].

### 1.4. Fingolimod

As the effects of fingolimod on the receptors are necessary for the formation of the circulatory system, it should not be used during pregnancy. The fingolimod needs two months for complete elimination; hence, contraception should be used during this time to prevent any potential risk to the fetus [53]. Karlsson *et al*. evaluated the effect of fingolimod on pregnancy outcomes among women who received it before six months and/or conception [54]. They revealed that 28 live births and 20 elective terminations out of 66 total pregnancies after exposure to the drug were recorded. While nine spontaneous abortions, four terminations due to fetal abnormalities, and two fetal malformations occurred [54].

### 1.5. Mitoxantrone

Nowadays, treatment with mitoxantrone-a chemotherapy agent-is rarely administered for patients with MS [55]. Premature labor, fetal growth restriction, and amenorrhea have been reported following the mitoxantrone prescribed. For this reason, a negative pregnancy test is mandatory before each dose of mitoxantrone [55]. Also, due to its cardiotoxicity, the treatment regimen includes each dose at intervals of three months and a maximum of 11 doses. Although mitoxantrone crosses the placenta minimally, significant levels are secreted in breastfeeding [55]. Therefore, after completing the treatment, a 6-month interval is recommended between the last dose and pregnancy [55].

### 1.6. Natalizumab (NTZ)

The NTZ is a human monoclonal IgG4 antibody that could inhibit the cell adhesion protein 4-integrin [22]. Although NTZ could actively cross from the placenta during the second and third trimesters, it could not present into fetal circulation until the placenta is established at 13-14 weeks of gestation [56]. The rate of relapse, which typically occurs 12 to 16 weeks following the end of treatment, increases significantly with NTZ discontinuation. Landi *et al*. demonstrated that women who continued their NTZ therapy during pregnancy had a lower risk of MS relapses than those whose treatment was stopped at an earlier stage of pregnancy or before conception [56]. Based on the Association of British Neurologists guidelines, it suggested that NTZ was discontinued after 34 weeks of gestation and resumed as soon as possible after delivery. Indeed, within 8 to 12 weeks of the last dose, NTZ should be restarted to avoid MS relapses [57].

Regarding the Tysabri Pregnancy Exposure Registry, the rate of fetal abnormalities and spontaneous abortions among women who received NTZ three months before conception or during pregnancy was higher than in the general population [58]. Another study showed that the administration of NTZ in the third trimester of pregnancy causes mild to moderate thrombocytopenia and anemia in 10 of 13 neonates. Additionally, all neonates' umbilical cord blood samples tested positive for NTZ [59]. Hence, it is required to evaluate newborn infants for thrombocytopenia and anemia in the cases of NTZ administered during pregnancy. The use of NTZ during pregnancy may be continued as it is a promising tactic [60].

### 1.7. Alemtuzumab

Alemtuzumab is another monoclonal antibody with a molecular weight of 150 kDa, a half-life of 4-5 days, and complete elimination in 30 days that interacts with the CD52 surface receptor on cells [61]. Premature labor, hypertension, low birth weight, and neurocognitive impairment are some fetal concerns associated with using alemtuzumab during pregnancy [62][63]. In contrast to monoclonal antibodies, alemtuzumab does not cross the placental during the first trimester. Nevertheless, it recommends using effective contraception for women under alemtuzumab therapy for at least four months after completion of treatment [61]. Oh *et al*. indicated that among 264 pregnancies exposed to alemtuzumab, 67% were live births without anomalies, 22% spontaneous abortions, 11% elective abortions, and one stillbirth [61]. Also, alemtuzumab is excreted in breast milk, like the majority of monoclonal antibodies; hence, breastfeeding is not advised [61].

### 1.8. Ocrelizumab

Ocrelizumab and rituximab are two human monoclonal anti-CD20 antibody treatments [64]. Despite little evidence for the safety of ocrelizumab during pregnancy, rituximab is the most effective anti-CD20 antibody treatment for MS [64]. In human studies as well as animal models, treatments with rituximab showed B-cell lymphocytopenia that persisted for up to six months after delivery [64]. Although a 12-month period following the final dose of rituximab is advised for trying pregnancy, Chakravarty *et al*. indicated that maternal exposure to rituximab in less than 12 months after the last dose, could lead to 22 preterm labor, one neonatal death (after six weeks), 11 cases of hematological abnormalities, four neonates with infections, and two congenital deformities [65]. Also, in animal studies, rituximab has been found in the milk of breastfeeding cynomolgus monkeys [64].

### 1.9. Cladribine

Cladribine is a purine nucleoside analog with a molecular weight of 285 Da that suppresses quickly proliferating cells and prevents DNA synthesis [52]. Since cladribine is teratogenic, it is encouraged that sexually active women continue using effective contraception for at least six months after the last dose [52]. Giovannoni *et al*. showed that outcomes of pregnancies in women exposed to cladribine during the at-risk time were similar to epidemiological statistics on the results of pregnancies in the general population [66]. Despite these findings, there is still very little information about cladribine; therefore, pregnancy and/or breastfeeding could be dangerous [66].

### 1.10. Dimethyl Fumarate (DMF)

DMF is the most recent first-line oral MS medication [67]. It could penetrate CNS in 25% of cases. Also, the previous evidence showed delayed ossification and embryo toxicity in pregnant rats with very high dosages [67]. In the Hellwig *et al*. [68] study, of 351 pregnancies, 277 were live births; 17 spontaneous abortions, including one molar and one ectopic pregnancy, were reported.

### 2. Symptomatic Treatments

Except for anticonvulsants, there is not enough data on the majority of MS symptomatic therapies to offer evidence-based recommendations for use during pregnancy [68]. Class B, C, or D risks are associated with commonly used MS symptomatic medications, such as baclofen, oxybutynin, amantadine, and clonazepam [68]. Symptomatic medications are often stopped before conception with an awareness of the potential functional effects; if continued, the smallest effective dose should be utilized for the shortest time [69].

## Postpartum Considerations

### Breastfeeding

Women who breastfed tended to experience fewer relapses. The results of a meta-analysis of 13 researchs on breastfeeding and MS relapse suggest that exclusive breastfeeding may lessen early postpartum relapses [70]. Returning to MS drugs should be considered carefully. IFN-β concentrations in breast milk are a tiny fraction of the maternal dose (0.006%), and GA is unlikely to be present in breast milk; however, it cannot be detected directly due to rapid degradation [70]. Also, its serum levels in the infants are so low because IgG antibodies, such as NTZ, pass into breast milk at considerably lower amounts than in serum and are mostly decomposition by the digestive system.

However, due to minimal digestion and delayed hepatic clearance, oral low molecular weight agents (such as fingolimod and DMF) are more likely to directly damage infants' neurological systems. Therefore, breastfeeding seems safe while a patient receives IFN-β, GA, NTZ, and other monoclonal antibodies. It may be required for women with highly active illnesses (i.e., pregnancies treated with NTZ, fingolimod, and/or cyclophosphamide) to stop breastfeeding and start DMT as soon as possible after birth [71].

### Prevention of Postpartum Relapses

Early postpartum relapse prevention may be achieved by restarting DMTs. Monthly intravenous methylprednisolone or Ig may reduce postpartum relapses, but more clinical studies are required to validate these effects [72].

## Conclusion

All patients with MS need to be educated regarding their disease and know that childbearing likely improves the long-term outlook for relapsing MS. Also, there is very little chance that MS will be passed on to future generations and could impact from none to minimal effects on fertility and pregnancy outcomes. Consequently, physicians involved with MS patients should be familiar with updated strategies for MS treatments for dealing with these pregnancy-related consequences.

## Conflict of interests

The authors declare that there is no conflict of interest regarding the publication of this article.
